# Tuberculosis in Patients with Anthracosis of Lung Underlying Mechanism or Superimposed Disease

**DOI:** 10.5812/kowsar.20741804.2247

**Published:** 2011-09-15

**Authors:** M H Mirsadraee, A K Asnashari, D M Attaran

**Affiliations:** 1 Internist and Subspecialty in Pulmonary Medicine, Islamic Azad University, Mashhad Branch, Mashhad, Iran

**Keywords:** Anthracosis, Anthracofibrosis, Tuberculosis, Acid fast bacilli, Caseating granuloma

## Abstract

**Background:**

Anthracosis is the black pigment discoloration of bronchi with an unknown cause which can cause bronchial destruction and deformity (anthracofibrosis). The objective of this study was to determine the frequency of tuberculosis in anthracosis and evaluate their association.

**Methods:**

One hundred and twenty subjects with bronchoscopy diagnosis of simple anthracosis, anthracofibrosis and non-anthracotic control groups entered this study. Demographic data and important clinical and radiological findings were recorded. Bronchial biopsy and bronchoalveolar lavage were performed for further cytopathological, acid-fast bacilli staining and culture in all cases.

**Results:**

Cough and dyspnea were significantly higher in anthracosis subjects. Radiological characteristic of tuberculosis such as upper lobe localization and cavity were not significantly higher in anthracosis subjects that suffer from tuberculosis. Laboratory test for tuberculosis showed positive acid fast bacilli in 17.5 and 25% and caseating granuloma in 20 and 17.5% of anthracosis and anthracofibrosis subjects. Adding the result of culture, the frequency of tuberculosis in both groups of anthracosis was 27.5% that was significantly more than nonanthracotic control group (Odd ratio= 6.15, CL=1.29<OR<40.06).

**Conclusion:**

Anthracosis and tuberculosis showed a significant association. Anti-tuberculosis therapy promised better treatment of anthracosis in subjects proved to suffer from tuberculosis.

## Introduction

Anthracosis of lung is the black discoloration of bronchial mucosal,[1] that its diagnosis is achievable by bronchoscopy. Sometimes anthracosis grows and occlude the lumen of bronchi; the condition that called anthracofibrosis.[2] Earlier reports showed that upper lobes were the most frequent involved location in lung.[1][3] According to this finding, tuberculosis as an underlying cause was suggested. In most of recent studies, frequency of tuberculosis was reported in a series of anthracotic subjects that underwent routine bronchoscopy but its frequency was not compared to a similar control group.

The objective of this study was to determine the presence of tuberculosis in subjects that demonstrated simple anthracosis and anthracofibrosis during routine bronchoscopy with a prospective controlled study design.

## Materials and Methods

All patients who underwent a flexible bronchoscopy for various indications in two university hospitals during the year of 2006-2008 were enrolled. The patients were divided into three groups: subjects with simple anthracosis, anthracofibrosis and nonanthracotic. Demographic data, history of exposure to smoke and important clinical findings were recorded. During bronchoscopy, subjects with superficial nondeforming black discoloration were classified as simple anthracosis and subjects with black discoloration that caused deformity or stenosis of bronchus were classified as anthracofibrosis. Control group was subjects without any black discoloration in bronchial tree. Bronchial lavage was obtained for cytology, acid-fast bacilli (AFB) and culture. Biopsy was taken for histopathology examination when infiltration in bronchial surface was observed or according to computed tomography transbronchial lung biopsy was taken from suspicious location.

This study was approved by Ethical Committee of Medical School of Islamic Azad University of Mashhad. Written consent was given from all patients. A sample size of 40 for each group was calculated as sufficient for a 0.05 alpha error and 80% potency on the basis of prevalence of anthracosis (22%).[3] Normal distribution of the data was checked for age by using Kolmogerov Smirnof test. Positive culture of bronchial lavage and/or histopathology consistent with tuberculosis was gold standard for diagnosis of tuberculosis. Chi Square test (X(2)) and 95% confidence limit (95% CI) were used to evaluate the significance of difference between the groups. EPI INFO 2003 and SPSS 14 software were used for statistical analysis. Significance was accepted at p<0.05.

## Results

Female to male ratio in whole group was 1:1, but in anthracosis groups female to male ratio was 1.33:1 that was significantly more than the control group (0.6, p=0.042). In tuberculosis confirmed subjects female to male ratio was 1.4:1 that was not significantly different from non-tuberculosis subjects. The mean age of anthracofibrosis and simple anthracosis groups (69±9.2 and 68±16.8 years respectively) were significantly higher than the control non-anthracotic group (55±19.3 years, p=0.001 and p=0.002 respectively).

Cigarette smoking was observed in 17% of anthracosis and 14% of tuberculosis subjects that in anthracosis group was significantly less than the control group (27%) (p=0.048). Baking the bread in rustic household oven was detected in 27 female subjects (54% of all female subjects) that were significantly more than male individuals (2, 7%, p=0.001). From these female subjects, 23 were anthracosis subjects and 4 were non-anthracotic, that made the difference significant (p=0.004, odd ratio= 4.8, CL=1.43<OR<20.54).

The most frequent clinical findings were shown in Table 1. Frequency of cough and dyspnea were significantly higher in tuberculosis group than anthracosis groups (p=0.023 and p=0.031 respectively). The difference for other symptoms was not significant. The results of laboratory tests for tuberculosis of specimen from bronchial lavage and bronchial biopsy were shown in Table 2. Positive smear for acid fast bacilli and caseating granuloma were significantly higher in anthracosis groups. Adding the result of culture, final diagnosis of tuberculosis was confirmed in 22 out of 80 anthracosis subjects (27.5%) that were significantly more than the control group (p=0.0003, odd ratio= 6.15, CL=1.29<OR<40.06).

**Table 1:** Comparison of demographic and clinical findings between anthracotic subjects and control nonanthracotic group

	**Simple anthracosis (%)**	**Anthracofibrosis (%)**	**Tuberculosis (%)**
Cigarette smoking	15	20	14
Bakery	44^a^	41^a^	15
Cough	86	75	91 a
Dyspnea	72	77	87 a
Sputum	37	32	44
Hemoptysis	20	16	13

^a^ P<0.05

**Table 2:** Comparison of laboratory and some radiological findings of tuberculosis between anthracotic subjects and control non-anthracotic group.

	**Simple anthracosis No (%)**	**Anthracofibrosis No (%)**	** Nonanthracotic No (%)**	**Total No (%)**
Acid fast bacilli a	7 (17.5)	10 (25)	1 (2.5)	18 (15)
Tubeculosis Granuloma a	8 (20)	7 (17.5)	1 (2.5)	16 (13.4)
Tuberculosis a	10 (25)	12 (30)	2 (5)	24 (20)
Upper lobe localization a	29 (82)	26 (81)	12 (30)	67 (55)
Cavity	2 (5.7)	1 (3)	5 (12)	8 (5)

^a^ P<0.05

Upper lobe localization was also more frequent in both anthracosis groups (p=0.0001). During bronchoscopy, 13 (24%) subjects with anthracosis were located in upper lobe bronchi. This finding was not correlated with final diagnosis of tuberculosis (p=0.6). Cavity was more frequent in nonanthracotic control group (12%) but the difference was not significant.

Follow up of tuberculosis subjects either with anthracosis or non-anthracosis for at least six months revealed significant improvement of clinical findings with anti-tuberculosis therapy (classical 6 months therapy). All patients were controlled except for some seasonal exacerbations.

## Discussion

The result of this study showed that tuberculosis was presented in 27.5% of anthracosis patients that was significantly higher than non-anthracotic control group. The odd ration for tuberculosis in these subjects was 6.15. Cough and dyspnea were significantly higher in anthracosis subjects but these symptoms were usually related to anthracosis by itself. Hemoptysis and radiological characteristic of tuberculosis such as upper lobe localization and cavity were not significantly higher in anthracosis subjects that suffered from tuberculosis; therefore these findings can not predict the presence of tuberculosis in anthracotic subjects. In contrast, laboratory tests including smear for acid fast bacilli, culture and histopathological findings such as caseating granuloma were successful in diagnosis of tuberculosis.

Some investigators diagnosed tuberculosis mostly by bacteriological tools. Towhidi et al.,[1] detected tuberculosis by direct smear for AFB in 96% and Törün et al.,[3] detected tuberculosis by culture in 100%. On the other hand, in some studies histopathological evaluation was the main stay for diagnosis of tuberculosis such as Hemmati et al.,[4] and Kim et al.,[5] that diagnosed tuberculosis by biopsy and histopathologic evaluation in 100% and 83% of their subjects respectively.

It seems that frequency of tuberculosis decreased by improving the health status of general population and governmental programs against tuberculosis. In Korea, earlier study by Chung et al.,[2] reported frequency as high as 60% of tuberculosis in anthracofibrosis, but recent studies in this country reported frequencies in a range of 20-34%.[6][7] One decade ago, in our region, frequency of tuberculosis was reported in 30% of anthracofibrosis subjects,1 but in present study and a large recent study in Iran by Amoli,8 its frequency declined to approximately 27%. In developed countries, tuberculosis in anthracofibrosis is even lower while Wynn et al. reported tuberculosis in only one anthracofibrosis subject in England.9 Overall frequency of tuberculosis in anthracofibrosis subjects during routine bronchoscopy in recent studies were in a range of 20-30%.[1][3][4][5][6][7][8][9] The type of Mycobacterium tuberculosis in anthracofibrosis was defined and was discovered that M. tuberculosis in anthracofibrosis is a reflection of this organism in the community.[10]

The underlying mechanism of anthracosis was not discovered up to now. We believe that tuberculosis is just a superimposition of infection on anthracofibrosis that predisposed by immune deficiency state that produced by anthracofibrosis and diagnosis of anthracosis is not equal to beginning anti tuberculosis treatment because more than 2/3 of our subjects did not approve to suffer from tuberculosis. Biomass or inorganic dust exposure are seem to be more substantial etiological factors for anthracofibrosis.[4][8][9] Anyway, treatment of tuberculosis helped us for better management and stopping the progressive course of anthracosis in anthracosis subjects. Therefore laboratory confirmation including acid fast bacilli smear, culture and histopathology of bronchial biopsy are required.

In conclusion, this study may end the controversies about presence of tuberculosis in anthracosis. Laboratory tests of tuberculosis are the only reliable methods for diagnosis of tuberculosis. Antituberculosis is a promising treatment for anthracotic subjects that the laboratory exam confirmed the involvement of lung by tuberculosis.
